# Perinatal Stroke and Cor Triatriatum Sinister: A Case Report

**DOI:** 10.7759/cureus.93211

**Published:** 2025-09-25

**Authors:** Inês Mazeda, Marisa Rodrigues, Jorge Moreira, Sandra Ramos

**Affiliations:** 1 Pediatrics and Neonatology, Unidade Local de Saúde Póvoa de Varzim/Vila do Conde, Póvoa de Varzim, PRT; 2 Pediatric Cardiology, Unidade Local de Saúde de São João, Porto, PRT

**Keywords:** congenital heart defect, cor triatriatum sinister, hemiparesis, pediatric neurodevelopment, perinatal stroke

## Abstract

Cor triatriatum sinister (CTS) is a rare congenital heart defect characterized by a fibromuscular membrane dividing the left atrium. Though often asymptomatic in incomplete forms, CTS has been associated with thromboembolic events. We describe a case of a female child with a history of perinatal stroke presenting with right hemiparesis. Prenatal imaging suggested lateral ventricle asymmetry and localized hemorrhage. Postnatal development was mostly normal until gait abnormalities emerged at 18 months. Neuroimaging confirmed left-sided brain injury consistent with a past vascular insult. Cardiac evaluation revealed non-obstructive CTS with no signs of hemodynamic compromise. Although a direct causal relationship could not be confirmed, the identification of CTS in a cryptogenic perinatal stroke case raises the possibility that subtle atrial anomalies may contribute to fetal thromboembolic events. This case reinforces the importance of comprehensive cardiac evaluation in perinatal strokes and illustrates a favorable neurodevelopmental outcome with early multidisciplinary intervention.

## Introduction

Cor triatriatum, first described by Church in 1868 as a left atrium divided by an abnormal septum and later named “cor triatriatum” by Borst M in 1905 [1-3], is a rare congenital cardiac defect characterized by a fibromuscular membrane that separates the left atrium (cor triatriatum sinister, CTS) or, less commonly, the right atrium (cor triatriatum dexter, CTD) into two distinct chambers [4].

Symptom onset and disease severity are determined by the size and number of membrane orifices, as well as by the presence of associated defects and other cardiac anomalies. Severe forms are typically diagnosed in infancy, while milder cases may be identified only incidentally in adulthood [5].

In infants and newborns, the manifestations of CTS are usually secondary to a relatively narrow opening in the accessory membrane, resulting in increased proximal left atrial pressure and pulmonary congestion. Dyspnea may range from mild to severe, including neonatal respiratory distress, which carries a higher mortality risk [6]. In adults, CTS most frequently presents with exertional dyspnea, orthopnea, and palpitations. Symptoms related to congestive heart failure have been reported in up to 26.9% of patients, often associated with the development of supramitral inflow obstruction, mitral regurgitation, pulmonary hypertension, or atrial fibrillation [3,6]. Ischemic or thromboembolic events are also well documented in adults, whereas syncope has only been described in isolated cases [7].

CTS is found in approximately 0.1% to 0.4% of patients with congenital heart disease and is frequently associated with other cardiac malformations in up to 80% of pediatric patients [6].

Clinically, CTS often presents with symptoms resembling mitral stenosis, as both conditions are characterized by hemodynamic disturbances that impair left atrial emptying and increase pulmonary venous pressure [8]. The condition has also been associated with cardioembolic stroke, resulting from blood stasis in the left atrium, atrial fibrillation, or coexisting atrial septal defects or patent foramen ovale [9,10]. However, it remains an extremely rare manifestation in both adults and children.

We describe an unusual case of CTS diagnosed in a 3-year-old girl who presented with right-sided hemiparesis secondary to a perinatal stroke.

## Case presentation

We present a case of a female child followed since birth by pediatric care due to prenatal detection of unilateral ventriculomegaly. Prenatal ultrasounds performed at the 24th and 31st weeks of gestation revealed progressive enlargement of the left lateral ventricle. Fetal MRI confirmed asymmetry of the lateral ventricles with a focal hypointense area in the left germinal matrix on T2-weighted imaging, suggesting a probable localized hemorrhage.

Family history was unremarkable for thromboembolic, cardiac, neurological, or relevant genetic conditions. The pregnancy was closely monitored and uneventful. Delivery occurred at 36 weeks + 2 days of gestation via spontaneous vaginal delivery. The newborn had a birth weight of 2550 g, with Apgar scores of 8 and 10 at 1 and 5 minutes, respectively. No other perinatal complications were reported..

A transfontanellar cranial ultrasound performed at one month of age and repeated at three months demonstrated marked asymmetry of the lateral ventricles, with significant dilation of the left lateral ventricle (Levene index: 21 mm on the left versus 12 mm on the right), focal ectasia, and ballooning of the left fronto-parietal ventricular wall, suggestive of volume loss in the surrounding brain parenchyma.

Early postnatal development was considered typical. The child achieved independent walking at 14 months and demonstrated appropriate language acquisition for age. No hand preference was observed until 18 months, consistent with normal development. However, at 18 months, gait abnormalities were noticed, characterized by right-sided toe-walking, internal foot rotation, and mild claudication. In this context, she was referred to pediatric neurology for evaluation.

On neurological examination, upper limb tone, strength, and coordination were normal and symmetrical. In the lower limbs, there was mild right-sided spasticity with increased tone, predominantly affecting the distal musculature, along with slight contracture of the Achilles tendon. Subtle motor asymmetry was not evident at rest but became more pronounced during dynamic motor tasks, particularly running.

Brain MRI performed at 2 years of age demonstrated left lateral ventriculomegaly secondary to left frontal white matter loss, an area of gliosis with petechial hemorrhagic sequelae, widening of the caudothalamic groove, mild left thalamic volume loss, and Wallerian degeneration of the left corticospinal tract, findings consistent with a prenatal vascular insult, likely due to a germinal matrix hemorrhage.

Following this diagnosis, a hematological evaluation, including a thrombophilia panel for both the child and her parents, was performed. No significant abnormalities were identified.

As part of systemic evaluation, transthoracic echocardiography demonstrated an incomplete, non-obstructive cor triatriatum sinister, with otherwise normal cardiac anatomy and function, and no evidence of intracardiac shunts or hemodynamic compromise. Cardiology follow-up confirmed the absence of hemodynamic impairment, and no intervention was required.

**Figure 1:**
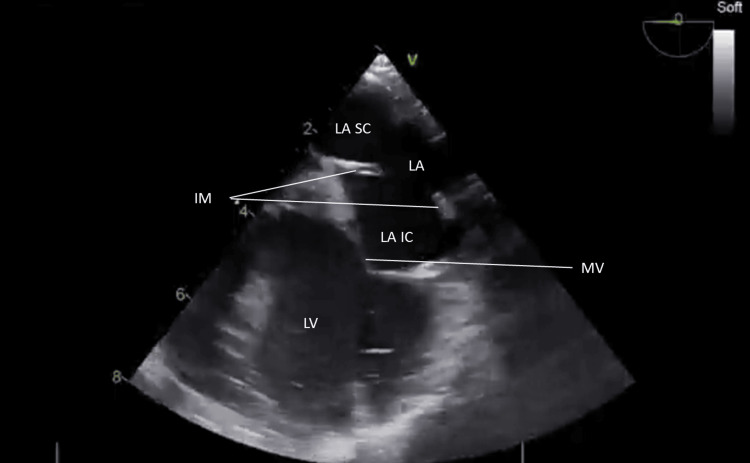
Transesophageal echocardiography at 0° shows an IM in the left atrium (LA), dividing it into superior (LA SC) and inferior compartments (LA IC), consistent with an incomplete, non-obstructive cor triatriatum sinister MV: Mitral valve; LV: Left ventricle; IM: incomplete membrane.

**Figure 2:**
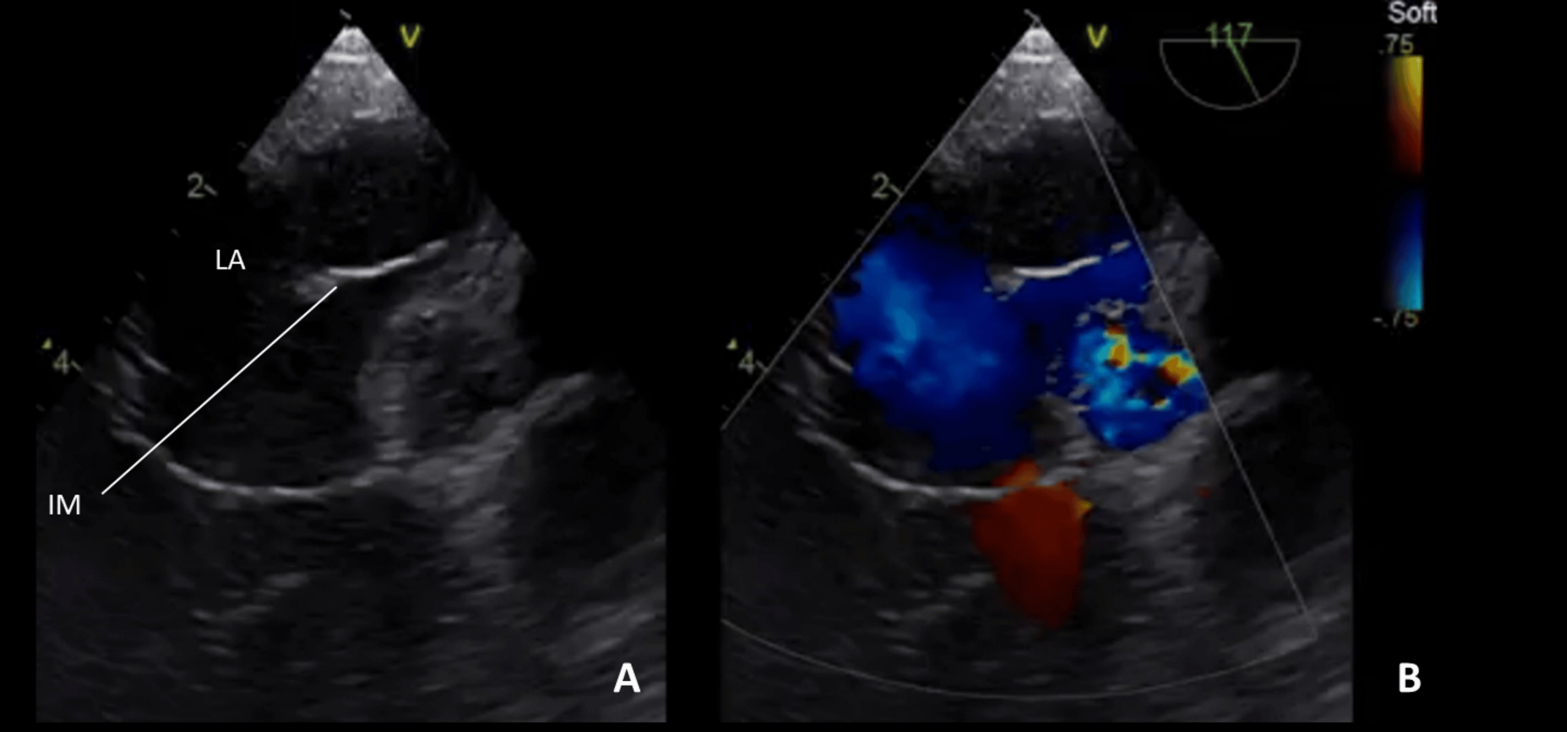
Cor triatriatum sinister, incomplete and non-obstructive. (A) Transesophageal echocardiography at 117° shows an incomplete membrane (IM) dividing the left atrium (LA) into two compartments. (B) Same view as in (A) with color Doppler demonstrating laminar flow between the compartments.

Transesophageal echocardiography further confirmed the diagnosis of cor triatriatum sinister, demonstrating the pathognomonic finding of the left atrial appendage arising from the true left atrial chamber, thereby distinguishing it from a supravalvular mitral membrane (SVMM). No interatrial communication, thrombus, or flow disturbance was identified (Figures 1-2).

The patient started physiotherapy twice a week, focusing on gait re-education and spasticity management. An ankle-foot orthosis was introduced to improve gait stability. Physical therapy sessions and extracurricular activities, including gymnastics and yoga, were later added to enhance coordination and balance.

Neurosensory evaluations, including ophthalmologic and otorhinolaryngologic assessments, revealed no significant abnormalities.

Over the following years, significant improvement was observed. The right hemiparesis became very mild and was barely noticeable during slow walking, becoming evident only during running. She maintained normal neurodevelopment, entered primary school without the need for special educational support, and actively participated in physical activities.

At six years of age, she remains under multidisciplinary follow-up, showing overall favorable neurological and motor developmental outcomes.

## Discussion

Perinatal stroke is an uncommon but significant cause of long-term neurological morbidity in neonates, particularly in preterm infants. With an estimated incidence of 38.6 per 100,000 live births, it accounts for a substantial proportion of congenital hemiparesis and cerebral palsy diagnosed in early childhood [10]. Its etiology is heterogeneous, encompassing maternal, placental, and fetal factors, yet in many cases remains unexplained and thus classified as cryptogenic [11].

Recent literature has increasingly emphasized the role of congenital cardiac anomalies in the pathogenesis of perinatal cerebral ischemia. While well-established risk factors include structural cardiac shunts such as patent foramen ovale, more subtle anomalies, particularly involving the left atrium, may be overlooked. CTS is typically diagnosed in infancy when obstruction leads to significant symptoms mimicking mitral stenosis. However, in non-obstructive or incomplete forms, CTS may be entirely asymptomatic and only incidentally discovered during cardiac imaging performed for unrelated reasons [12].

Despite the absence of hemodynamic compromise, it is conceivable that even non-obstructive CTS could disrupt intra-atrial flow, fostering blood stasis and predisposing to thrombus formation during fetal life. Altered pulmonary venous drainage and localized turbulence may further amplify this risk [6]. Although speculative, such mechanisms warrant consideration in cases of cryptogenic fetal stroke, especially when conventional prothrombotic or infectious etiologies are excluded.

In this case, thorough cardiac assessment, including transthoracic and transesophageal echocardiography, confirmed the presence of an incomplete, non-obstructive CTS with normal biventricular function, no septal defects, and no evidence of thrombus. While a direct causal link to the stroke could not be established, the co-occurrence raises the possibility of CTS as a contributing factor in an otherwise idiopathic event. This aligns with previous reports suggesting that even mild atrial anomalies may represent underrecognized contributors to neonatal ischemic injury [7].

Invasive hemodynamic assessment through cardiac catheterization can provide important information on left and right ventricular pressures, pulmonary artery pressure, and pulmonary vascular resistance. It may also help to exclude additional anomalies such as atrial septal defect, patent foramen ovale, or coronary anomalies, which could predispose to stroke or sudden cardiac death and are potentially amenable to surgical correction [3,6,13]. In our patient, cardiac catheterization was not performed because she remained asymptomatic, with normal transthoracic and transesophageal echocardiographic findings and no evidence of hemodynamic compromise.

From a prognostic standpoint, the neurodevelopmental outcome in children with unilateral fetal stroke is often favorable, particularly when identified early and supported by comprehensive rehabilitation. Early initiation of physiotherapy and occupational therapy has been shown to enhance motor recovery and functional independence, while preserved cognitive and language development may support full academic integration [14].

This case underscores the importance of maintaining a broad differential diagnosis in perinatal stroke and reinforces the value of detailed cardiac evaluation, even in the absence of overt cardiovascular symptoms. As non-obstructive CTS continues to be identified more frequently due to improved imaging resolution, further research is needed to clarify its role, if any, in fetal and neonatal cerebrovascular events.

In addition, CTS is frequently associated with other congenital cardiac anomalies, most notably atrial septal defects and anomalous pulmonary venous return, which may further increase the risk of cardioembolic events [7]. Other congenital cardiac lesions associated with CTS are summarized in Table 1 [6,12,15]. In symptomatic or obstructive cases, surgical resection of the fibromuscular membrane is the treatment of choice and is associated with excellent long-term outcomes [2,15-17]. In contrast, non-obstructive and asymptomatic cases, as in our patient, are often managed conservatively with regular cardiology follow-up [18]. The role of surgical or catheter-based interventions in preventing future stroke in non-obstructive CTS remains uncertain, highlighting the need for further research in this area.

**Table 1:** Congenital cardiac lesions associated with cor triatriatum sinister (CTS).

Associated congenital cardiac lesions
Atrial septal defect
Anomalous pulmonary venous return
Tetralogy of Fallot
Bicuspid aortic valve
Double-outlet right ventricle
Coarctation of the aorta
Persistent left superior vena cava with unroofed coronary sinus

## Conclusions

This case highlights the potential, albeit uncertain, association between CTS and perinatal stroke. While no direct causal relationship could be demonstrated, the coexistence of an incomplete, non-obstructive atrial membrane with a cryptogenic fetal vascular insult raises important considerations regarding the role of subtle cardiac anomalies in neonatal cerebrovascular disease. Comprehensive cardiac assessment should be encouraged in cases of unexplained perinatal stroke, even in the absence of overt cardiovascular symptoms, with transthoracic echocardiography as the first-line modality and transesophageal echocardiography for detailed visualization of the left atrium and interatrial septum. From a therapeutic perspective, surgical resection is indicated in symptomatic or obstructive cases of CTS, whereas conservative management with regular follow-up is appropriate in non-obstructive and asymptomatic forms, as in our patient. Finally, this report underscores the favorable prognosis that can be achieved through early recognition, timely rehabilitation, and multidisciplinary follow-up, ultimately supporting optimal neurodevelopmental outcomes.
